# Personality and Cognition: Sociability Negatively Predicts Shoal Size Discrimination Performance in Guppies

**DOI:** 10.3389/fpsyg.2017.01118

**Published:** 2017-06-30

**Authors:** Tyrone Lucon-Xiccato, Marco Dadda

**Affiliations:** Dipartimento di Psicologia Generale, Università degli Studi di PadovaPadova, Italy

**Keywords:** cognitive abilities, individual differences, personality, *Poecilia reticulata*, shoal choice

## Abstract

Evidence from a growing number of organisms suggests that individuals show consistent performance differences in cognitive tasks. According to empirical and theoretical studies, these cognitive differences might be at least partially related to personality. We tested this hypothesis in the guppy, *Poecilia reticulata*, by comparing individuals with different degree of sociability in the discrimination of shoals formed by a different number of conspecifics. We found that individual guppies show repeatability of sociability as expected for personality traits. Furthermore, individuals with higher sociability showed poorer shoal size discrimination performance and were less efficient in choosing the larger shoal compared to individuals with low sociability. As choosing the larger shoal is an important strategy of defense against predators for guppies, we discuss this relationship between personality and cognition in the light of its fitness consequences.

## Introduction

Human psychologists have long been interested in understanding why individuals show differential performance in cognitive tasks (Galton, 1869). Now, evidence of individual differences in cognitive performance begins to accumulate also in non-human mammals, birds and some fish species (reviewed in Thornton and Lukas, 2012; Guillette et al., 2016; Lucon-Xiccato and Bisazza, 2017). Yet, the causes of this variation are still poorly understood.

The most straightforward explanation for these findings is the presence of individual differences in the neural systems that support the cognitive aspects of task performance (Kanai and Rees, 2011); however, there is evidence that several other factors such as attention, motivation, experience, aging and stress might affect cognitive performance in both human and other animals (Dweck et al., 2004; Lavie, 2005; Derakshan and Eysenck, 2009; Lupien et al., 2009; Thornton and Lukas, 2012; Lucon-Xiccato and Bisazza, 2017). Recently, personality has been recognized as a factor that might be related to cognitive performance in several species (Carere and Locurto, 2011; Sih and Del Giudice, 2012). Animals often show consistent individual differences in behaviors such as sociability, boldness, and exploratory tendency often referred as personality (reviewed in Dingemanse and Réale, 2005). Several authors have hypothesized the existence of a relationship between individual differences in cognitive abilities and personality (Carere and Locurto, 2011; Sih and Del Giudice, 2012). Though empirical tests of this hypothesis are still scarce, there is support from several studies (reviewed in Sih and Del Giudice, 2012; Lucon-Xiccato and Bisazza, 2017).

Individual differences in cognition have been often related to fitness (Keagy et al., 2009; Cauchard et al., 2013; Smith et al., 2015); thus, the different cognitive performance showed by individuals with different personality types might have important consequences. However, studies on the relationship between personality and cognition have not usually measured individual fitness and have been performed using laboratory conditioning procedures which are difficult to link to the cognitive tasks faced by animals in their natural environment. For example, Budaev and Zhuikov (1998) tested guppies, *Poecilia reticulata*, with different personality types using an avoidance learning task in a shuttle box; in a study on cavies, *Cavia aperea*, subjects had to learn to access a food reward hidden inside a plastic cylinder (Guenther et al., 2014). To understand the fitness consequences of the relationship between cognition and personality, and ultimately understand how this relationship evolves, we arguably need to focus on cognitive tasks similar to the ones performed by animals in their natural environment (see Thornton et al., 2014).

Here, we investigated the association between performance in a cognitive task and a personality trait in a fish species, the guppy. In particular, our goal was to test the hypothesis that individual guppies’ shoal size discrimination performance is related to individual variation in sociability. We chose to focus on the discrimination of shoal size for two reasons. First, this cognitive task mimics a natural situation in which the choice accuracy due to individuals’ cognitive abilities may have important fitness consequences. Indeed, joining the larger available shoal is one of the main antipredator strategies of guppies and other social fish as it dilutes individual risk (Magurran and Seghers, 1994; Krause and Godin, 1995). Consequently, guppies have been selected for refined shoal size discrimination abilities (reviewed in Agrillo et al., 2017). The second reason for focusing on the discrimination of shoal size is that this is the only cognitive task in which guppies have been tested for individual differences in performance. Thought guppies show an average preference for the larger shoal among options (Agrillo et al., 2012), previous studies have demonstrated that when the numerical difference between two shoals is subtle (e.g., four versus six fish), some guppies consistently achieve better performance than others (Miletto Petrazzini and Agrillo, 2016; Lucon-Xiccato and Dadda, 2017). Regarding sociability, it is considered a personality trait in guppies and could be associated to shoal size discrimination ability (Magurran and Seghers, 1991; Brown and Irving, 2013; Irving and Brown, 2013; Cattelan et al., 2017).

To achieve our goal, we performed two experiments. In a preliminary experiment (experiment 1), we assessed whether sociability is a personality trait in our guppy population and whether our sociability test (an octagonal mirror test) provides a valid measure of this trait (Burns, 2008; Beckmann and Biro, 2013; Cattelan et al., 2017). We tested individual guppies twice in the mirror test to evaluate repeatability. A significant repeatability of the score across the two trials would indicate that sociability is a personality trait in our population and that our test allows to detect individual variation for this trait.

In experiment 2, we addressed our main hypothesis by correlating the sociability of guppies measured with the mirror test to the variance in performance in a four versus six fish discrimination task. Sociability could affect shoal size discrimination performance in multiple ways, making it possible to formulate different predictions on the results of this experiment. For example, one might expect a positive correlation between the two traits because highly social individuals might be more motivated to join the larger shoal (Irving and Brown, 2013); on the other hand, a negative relationship could equally subsist because highly social individuals may show reduced choosiness between social groups (Cote et al., 2012). Given the scarcity of previous studies on this topic, it was difficult to predict the direction of the correlation in our experiment and we adopted an explorative approach.

## Materials and Methods

### Subjects

We used female domestic guppies of a stock bred in our laboratory since 2012. In experiment 1, we tested 20 subjects, whereas in experiment 2, we tested 42 subjects. To minimize differences between the subjects, all the fish used in one experiment were of the same age class (7 months in experiment 1 and 8 months in experiment 2). Furthermore, before the experiments, we moved all the fish in the same glass aquaria; by visually comparing the individuals, we chose only subjects with the same size. Before the experiments, we maintained small groups of guppies (approximately 20 individuals) in 70-L glass aquaria enriched with natural vegetation and natural gravel. The aquaria were also provided with water filters and fluorescent lamps (illumination from 0730 to 1930 h). Water temperature was kept at 26 ± 1°C. We fed the guppies *ad libitum* using alternate flakes (Super Hi Group, Ovada, Italy) and live *Artemia salina* nauplii.

### Ethics Statement

This study was carried out in accordance with the recommendations of law of our country (Italy, D.L. 4 Marzo 2014, n. 26). The protocol was approved by the Ethical Committee of Università di Padova (protocol n. 32/2015).

### Experimental Design

In experiment 1, we tested the repeatability of individual differences in sociability with the mirror test. For this purpose, we housed 20 female guppies individually in aquaria similar to the ones used for maintenance before the experiments but smaller (20 × 50 × 30 cm; water depth 20 cm). We provided gravel bottom, plants, and water filter, and eight guppies as social companions (two adult males and six immatures). After 7 days of habituation to the novel aquarium, we tested each subject in the mirror test. Then, we moved the subjects back to their individual aquarium for 3 days and, after this interval, we re-tested the subjects in the mirror test to correlate the scores of the two trials.

In experiment 2, we tested for the presence of a correlation between shoal size discrimination performance and sociability. Guppies collected from the maintenance tanks firstly underwent the mirror test. After completion of the mirror test, guppies underwent the shoal size discrimination task. We did not randomize the test order to increase statistical power (Bell, 2013).

### Experimental Apparatuses and Procedures

#### Mirror Test

**FIGURE 1:**
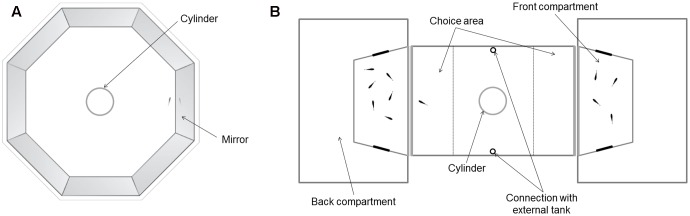
View from above of the apparatus used for **(A)** the mirror test and **(B)** the shoal size discrimination task.

The mirror test was performed following a well-established procedure (Dadda et al., 2007, 2015). We placed the fish into a transparent cylinder (Ø 8 cm) in the middle of an octagonal tank with mirrored walls (mirrors’ size: 28 cm × 37 cm; water depth: 10 cm; **Figure 1A**). The tank was lit by four 15-w fluorescent lamps. We raised the cylinder after a 2-min settling period, leaving the guppy free to swim for 10 min, and we recorded the test from above. Since guppies perceive their mirror image as a real conspecific, they tend to swim in close proximity of the mirror (Dadda et al., 2007). Using the video recordings, we measured the percentage of time spent within 1 cm from the mirror as a proxy of sociability (Dadda et al., 2015). Individuals with high sociability are expected to spend more time close to the mirror.

#### Shoal Size Discrimination Task

The shoal size discrimination test was performed using our three-tank apparatus described in details elsewhere (Dadda et al., 2015; Lucon-Xiccato et al., 2016a,b; Lucon-Xiccato and Dadda, 2017; **Figure 1B**). Briefly, the central tank (36 cm × 60 cm × 35 cm; water depth: 18 cm) had long walls and bottom covered with green plastics, and housed the subject during the test. To provide conspecifics’ olfactory cues to the subject, the central tank was connected to an external 400-L tank with a large population of guppies (approximately 50 individuals of both sexes) thanks to two pumps (water flow: 1.5 L/min). The two lateral tanks (36 cm × 60 cm × 35 cm; water depth: 18 cm) housed a shoal of 14 females each. These tanks were subdivided into a back (40 cm × 18 cm) and a front compartment (40 cm × 22 cm) connected by lateral corridors. The front compartment was visible from the central tank and housed the stimulus fish during the test. A 15-w fluorescent lamp placed above the front compartment lid the stimuli and provided also indirect illumination to the subject in the central tank. Half an hour before the experiment, we blocked a shoal of 4 and a shoal of 6 female guppies in the two front compartments. Shoals made of 4–6 individuals are commonly observed in natural guppy populations (Croft et al., 2003). Furthermore, the discrimination between 4 and 6 fish is close to the threshold of guppies’ numerical discrimination ability, as only some individuals are able to fully achieve it (Lucon-Xiccato et al., 2016b; Miletto Petrazzini and Agrillo, 2016); thus, the difficulty of this discrimination was expected to favor detection of individual differences. The position of the larger shoal was counterbalanced between tests. After testing each subject, we substituted the guppies of the stimulus shoals with other guppies housed in the lateral tanks. To start the trial, we moved the subject collected from the mirror test apparatus into a small transparent cylinder (Ø 8 cm) in the middle of the central tank for a 2-min setting period. Then, we raised the cylinder and left the subject free to swim in the central tank for 28 min. Being in a unfamiliar tank with no cover, guppies were expected to show antipredator behavior and join the larger available shoal. Following previous studies on shoal size discrimination abilities in guppies and other fish species (Agrillo et al., 2008; Bisazza et al., 2014; Dadda et al., 2015; Miletto Petrazzini and Agrillo, 2016), we measured time spent by the subject in a choice area within 11 cm from each stimulus shoal. We calculated the preference for the larger shoal as: time spent close to the larger shoal/(time spent close to the larger shoal + time spent close to the smaller shoal) × 100.

### Statistical Analysis

Statistical analysis was performed in R version 3.4.0 (The R Foundation for Statistical Computing, Vienna, Austria)^1^. Statistical significance was set at *P* = 0.5. Descriptive statistics reported in the text are mean ± standard deviation. We used paired-samples *t*-test to compare the sociability scores of the two mirror test trials in experiment 1 and one-sample *t*-test to assess whether the preference for the larger shoal in experiment 2 was greater than chance (50%). Effect sizes for the *t*-tests (Cohen’s *d*) were calculated using the ‘powerAnalysis’ R package. In experiment 1, repeatability (*R*) between the two trials with the mirror test was computed from generalized linear mixed-effects model with Gaussian error distribution fitted by restricted maximum likelihood (Nakagawa and Schielzeth, 2010) using the ‘rptR’ R package. In experiment 2, we tested for a correlation between shoal size discrimination performance and sociability using Pearson correlation.

## Results

### Experiment 1: Repeatability of Individual Differences in Sociability

**FIGURE 2:**
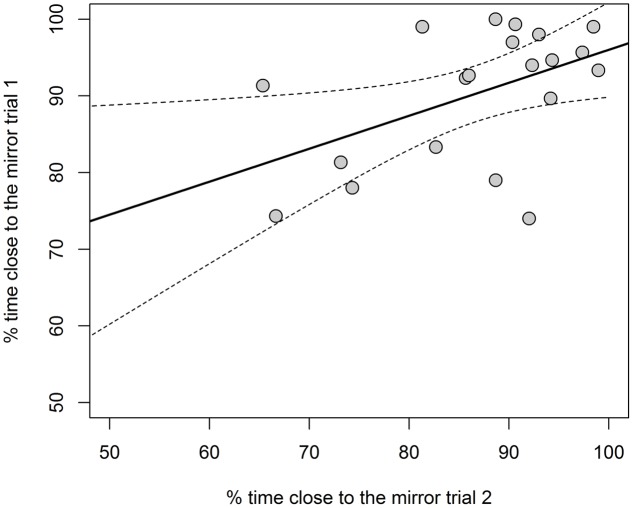
Results of experiment 1. Scatterplot of the sociability (% time close to the mirror) measured in the first versus the second trial of the mirror test. Continuous lines are predicted values and dashed lines are 95% C.I. of linear regression.

In the first trial of the mirror test, guppies spent 90.30 ± 8.71% time within 1 cm from the mirror; in the second trial, guppies spent 86.71 ± 9.98% time within 1 cm from the mirror. There was no significant difference between the average sociability scores of the two trials in the mirror test (paired-samples *t*-test: *t*_19_ = 1.695, *P* = 0.106, *r* = 0.188, Cohen’s *d* = 0.389). There was significant repeatability of sociability across the two trials with the mirror test (*R* = 0.488 *P* = 0.0098; **Figure 2**).

### Experiment 2: Correlation between Shoal Size Discrimination Performance and Sociability

**FIGURE 3:**
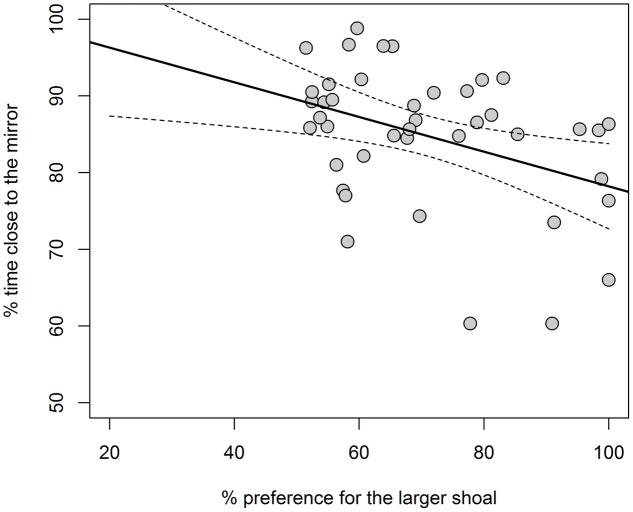
Results of experiment 2. Scatterplot of the sociability (% time close to the mirror) versus shoal size discrimination performance (% preference for the larger shoal). Continuous lines are predicted values and dashed lines are 95% C.I. of linear regression.

In the mirror test of experiment 2, guppies spent 84.81 ± 9.06% time within 1 cm from the mirror, indicating social attraction for the mirror image. In the shoal size discrimination test, guppies spent 70.84 ± 15.84% time close to the larger shoal, a preference that was significantly greater than chance (one-sample *t*-test against 50%: *t*_41_ = 8.526, *P* < 0.0001, Cohen’s *d* = 1.332). We found a negative correlation between sociability and shoal size discrimination performance (Pearson’s correlation: *r*_40_ = -0.40, *P* = 0.0096; **Figure 3**).

## Discussion

Our first experiment reveals that the sociability score of the mirror test was repeatable across multiple trials, suggesting that it measures the consistent individual differences in sociability (i.e., personality) which have been previously observed in guppies (Brown and Irving, 2013; Irving and Brown, 2013; Cattelan et al., 2017). In our second experiment, we found that individual guppies showing high sociability in the mirror test showed reduced abilities to discriminate between two shoals with a subtle size difference (four and six fish). Taken together, results of the two experiments suggest that guppies’ sociability, which is a personality trait in this species, is associated with shoal size discrimination performance. This finding aligns with growing evidence of a relationship between individual differences in cognition and personality in fish and other groups (Sih and Del Giudice, 2012; Lucon-Xiccato and Bisazza, 2017). It is worth noting that our study demonstrates the existence of such relationship in a spontaneous task mimicking fitness-related decisions faced by animals in their natural environment.

Despite the growing evidence of a correlation between individual cognitive differences and personality, it is still difficult to understand the mechanism underlying this relationship. Personality traits might affect cognitive performance in two different ways, which lead to different hypotheses and predictions on the direction of the relationship. First, personality might be linked to the cognitive ability of individuals because the association is advantageous (Guillette et al., 2016). For example, individuals that explore faster have a high likelihood to encounter novel situations; thus, they arguably benefit from having the ability to learn rapidly (Sih and Del Giudice, 2012). In these situations, selection could favor the association of the two traits via mechanisms such as genetic association and pleiotropy. Following this idea, one could expect two possible outcomes for our second experiment. On the one hand, one could expect better shoal size discrimination performance in individuals with high sociability because, at the inter-specific level, sociability has been often associated with enhanced cognitive abilities (Dunbar and Shultz, 2007). However, our results did not align with this hypothesis. On the other hand, individual guppies with low sociability likely spend more time alone as observed in another poeciliid fish (Cote et al., 2012); thus, individuals with low sociability are likely required to solve the problem of choosing between different shoals more often than social individuals that rarely separate from their social group. In line with this idea, it has been observed that male guppies, which are less social than females, are more likely to change social group compared to females (Griffiths and Magurran, 1998). As a consequence, less sociable guppies might have been selected for enhanced shoal size discrimination abilities, an explanation consistent with the result of the present study.

Personality might affect cognition also in another way. Certain personality types are expected to increase the probability of solving a cognitive task without implying an enhancement of cognitive abilities *per se* (Guillette et al., 2016). For example, fast explorers encounter a specific problem more often and thus are more likely to find its solution. Following this idea, one could expect better shoal discrimination performance in highly social individuals because they are more motivated to join the larger shoal or because they pay more attention to conspecifics, as shown by a previous study on guppies (Trompf and Brown, 2014). However, this hypothesis seems to conflict with our result. Alternatively, it is possible that individuals with different sociability differ in choosiness. In the western mosquitofish, *Gambusia affinis*, more social individuals showed reduced choosiness between shoals (Cote et al., 2012). It is possible that in our experiment the differential performance of individual guppies in the shoal size discrimination task was due to individual differences in choosiness. Studies using different approaches would help to disentangle these different mechanisms. For example, it may be useful to subtly delineate the behavior of the individuals in social context using social networks and direct observations in natural settings and to correlate the different behaviors to the ability in discriminating shoal size.

Whatever the underlying mechanism, our finding provides novel insights on the possible fitness consequences of the relationship between cognition and personality. At the intraspecific level, high cognitive performance is often associated to greater fitness. For instance, male rose bitterlings with better spatial learning performance are more efficient in competing to fertilize female eggs (Smith et al., 2015) and in two bird species problem solving performance positively predicts reproductive success (Keagy et al., 2009; Cauchard et al., 2013). In most of the studies on the relationship between cognition and personality, it was difficult to draw conclusions on fitness consequences; conversely, this was possible in our study because we used a task naturally performed by guppies (Croft et al., 2003). Shoaling with larger group is one of the main antipredator strategies of social fish (Magurran and Pitcher, 1987; Hager and Helfman, 1991; Krause and Godin, 1994; Magurran and Seghers, 1994). Indeed, being in a large group dilutes individual risk (Krause and Ruxton, 2002), increases vigilance (Magurran et al., 1985) and decreases predator hunting success (Krause and Godin, 1995). Thus, the relationship observed in our study is likely to have a profound impact on guppies’ fitness: less social guppies are expected to show greater survival under high predation risk.

Given this situation, one crucial point remains unclear. Selection due to predators is expected to favor individuals with low sociability and to rapidly deplete individual variation in shoal size discrimination ability. One possible general explanation for the absence of this phenomenon is that social and non-social guppies are differentially favored in different environments and that spatio-temporal fluctuations in predation risk have allowed the maintenance of the different phenotypes (Bell, 2010). It is, however, equally possible that other co-occurring selective pressures acting on personality are responsible for this apparent paradox (reviewed in Dingemanse and Réale, 2005; Biro and Stamps, 2008; Schuett et al., 2010). Beside these general explanations, the literature suggests further explanations that might specifically apply to this study case. Highly social guppies might show better performance in other cognitive tasks that balances the costs of the reduced shoal size discrimination ability. For example, shy rainbow trout, *Oncorhynchus mykiss*, show better memory for predators than bold trout (Brown et al., 2013). Since guppies with low sociability are often shier (Irving and Brown, 2013), they might possess enhanced predator recognition memory in parallel to what observed in trout and rely on this ability to deal with predation risk. It has also been show that guppies with reduced shoal size discrimination ability (i.e., the more social individuals) perform better in a task that requires them to judge the size of two food items and to select the larger, more profitable item (Lucon-Xiccato and Dadda, 2017); thus, the benefits resulting from enhanced accuracy in foraging decisions might balance reduced shoal size discrimination performance in highly social guppies. Other possible advantages of highly social guppies do not regard cognitive abilities. For example, it is possible that highly social guppies suffer less competition during foraging because they often choose the smaller group (Hoare et al., 2004) or that they are generally better foragers because they are more likely to exploit social information on foraging patches (Trompf and Brown, 2014).

## Conclusion

The results of this study suggest that the relation between individual variation in cognition and personality may have relevant fitness consequences, and indicate that experiments on cognitive tasks naturally performed by the species might be useful in understanding the evolutionary causes and consequences of this interaction.

## Author Contributions

TL-X and MD designed the study; MD collected the data; TL-X and MD analyzed the data and wrote the manuscript.

## Conflict of Interest Statement

The authors declare that the research was conducted in the absence of any commercial or financial relationships that could be construed as a potential conflict of interest.

## Footnotes

1 http://www.r-project.org
